# Recurrence of rectal cancer on the pelvic sidewall after lateral lymph node dissection

**DOI:** 10.1007/s00384-024-04650-7

**Published:** 2024-05-28

**Authors:** Misato Takao, Kazushige Kawai, Daisuke Nakano, Akira Dejima, Sakiko Nakamori, Soichiro Natsume, Ichiro Ise, Hiroki Kato, Tatsuro Yamaguchi

**Affiliations:** 1 Department of Surgery, Tokyo Metropolitan Cancer and Infectious Diseases Center Komagome Hospital, 3-18-22, Honkomagome, Bunkyo-ku, Tokyo, 113-0021 Japan; 2 Department of Clinical Genetics, Tokyo Metropolitan Cancer and Infectious Diseases Center Komagome Hospital, Tokyo, Japan

**Keywords:** Rectal cancer, Lateral lymph nodes, Recurrence, Local recurrence

## Abstract

**Purpose:**

Although lateral lymph node dissection has been performed to prevent lateral pelvic recurrence in locally advanced lower rectal cancer, the incidence of lateral pelvic recurrence after this procedure has not been investigated. Therefore, this study aimed to investigate the long-term outcomes of patients who underwent lateral pelvic lymph node dissection, with a particular focus on recurrence patterns.

**Methods:**

This was a retrospective study conducted at a single high-volume cancer center in Japan. A total of 493 consecutive patients with stage II-III rectal cancer who underwent lateral lymph node dissection between January 2005 and August 2022 were included. The primary outcome measures included patterns of recurrence, overall survival, and relapse-free survival. Patterns of recurrence were categorized as lateral or central pelvic.

**Results:**

Among patients who underwent lateral lymph node dissection, 18.1% had pathologically positive lateral lymph node metastasis. Lateral pelvic recurrence occurred in 5.5% of patients after surgery. Multivariate analysis identified age > 75 years, lateral lymph node metastasis, and adjuvant chemotherapy as independent risk factors for lateral pelvic recurrence. Evaluation of the recurrence rate by dissection area revealed approximately 1% of recurrences in each area after dissection.

**Conclusion:**

We demonstrated the prognostic outcome and limitations of lateral lymph node dissection for patients with advanced lower rectal cancer, focusing on the incidence of recurrence in the lateral area after the dissection. Our study emphasizes the clinical importance of lateral lymph node dissection, which is an essential technique that surgeons should acquire.

## Introduction

Local recurrence reportedly develops in 4–15% of patients with locally advanced rectal cancer who undergo R0 tumor resection [1–4]. Preoperative chemoradiotherapy (CRT) has been widely used to reduce local recurrence, and favorable local control has been reported [5–7]. However, in some cases with lateral lymph node (LLN) metastasis, CRT has been proven insufficient for local control, and some studies have demonstrated that the addition of lateral lymph node dissection (LLND) to conventional total mesorectal excision (TME), even in cases of preoperative CRT, contributes to better local control [8, 9].

LLND, a systemic dissection of lymph nodes in the pelvic sidewall, such as those in the obturator or internal iliac areas, has been performed to reduce local recurrence as the standard therapy for locally advanced rectal cancer in Japan [10]. The concept and technique of LLND were originally developed in Western countries [11, 12]. However, in 1959, Stearns and Deddish reported discouraging results, noting high morbidity and mortality without oncological benefit [13]. Since then, LLND has been regarded as an ineffective procedure and was abandoned in Western countries. However, in recent years, the number of institutions performing LLND on patients with suspected LLN metastasis has increased even in Western countries, underscoring the growing importance of understanding the prognostic outcome after LLND. Nevertheless, the detailed incidence of lateral pelvic recurrence (LPR) after LLND has not yet been fully investigated.

In the present study, we aimed to investigate the long-term outcomes of patients who underwent LLND using a large retrospective cohort, with a particular focus on recurrence patterns.

## Materials and methods

### Patients

The medical records of 493 consecutive patients with clinical stage II–III rectal cancer who underwent TME and LLND between January 2005 and August 2022 were retrospectively reviewed. Tumor progression, location, and the presence of LLN metastasis were evaluated using imaging modalities such as colonoscopy, computed tomography (CT), and magnetic resonance imaging in some cases. The clinicopathological features of the patients were determined from their medical records, and tumor features and stages were classified according to the TNM classification system [14]. Previous cases were reclassified according to the latest edition of the TNM classification, the eighth edition. In cases where the lower edge of the tumor was located below the peritoneal reflection and invasion beyond the proper muscle was suspected, TME plus bilateral LLND was performed regardless of the presence or absence of lymph node metastases in the preoperative diagnosis. Unilateral lymph node dissection was performed if the distal edge of the tumor was above peritoneal reflection (PR) but LLN metastasis was suspected. There were also cases in the early years of the study in which unilateral dissection was performed based on the predominant location of the tumor, due to old age and poor general medical condition (e.g., right-sided dissection in the case of right wall lesions). This retrospective study was approved by the Institutional Ethics Committee of Komagome Hospital (no.3130), and consent was obtained from the participating patients in the form of an opt-out.

### Procedure

This was a retrospective cohort study. The collected variables included age, sex, body mass index, Information on surgical procedures, tumor size, histological subtype, stage, chemotherapy, recurrence site, and survival time. Only data available for all the cohort were statistically analyzed to reduce the impact of information bias. The endpoint of this study was LPR. All the patients underwent radical surgery, including TME with bilateral or unilateral LLND without preoperative treatment. We defined LLND as complete dissection of the lymph nodes, at least in the internal iliac and obturator areas. The final stage was determined by examining the resected specimens according to the eight edition of the TNM classification for colorectal cancer [14]. Adjuvant chemotherapy was administered to patients with pathological stage III disease. Recurrence was monitored by regular examinations, including office visits and tumor marker assays every 3–6 months, colonoscopy every 1–2 years, and CT every 6 months. Postoperative surveillance was performed for 5 years according to the Japanese Society for Cancer of the Colon and Rectum (JSCCR) guidelines [10]. Local recurrence was diagnosed radiologically or histologically. Local recurrence was categorized into LPR and central pelvic recurrences (CPR).

### Statistical analysis

Continuous variables were presented as means and standard deviations or medians and ranges, whereas categorical variables were presented as frequencies and percentages. The Fisher’s exact test or *t*-test was used to compare differences between groups, as appropriate. Overall survival (OS) and relapse-free survival (RFS) were analyzed using the Kaplan–Meier method and compared between the groups using log-rank tests. RFS was defined as the time interval between LLND and disease recurrence or death. OS was defined as the interval between LLND and death from any cause. Univariate and multivariate analyses of the lateral pelvic wall recurrence were performed using the Cox proportional hazards model. Multivariate analysis was performed using all variables with a *p*-value of < 0.10 in univariate analysis. Statistical significance was set at *p* < 0.05. All statistical analyses were performed using EZR (Saitama Medical Center, Jichi Medical University, Japan), a graphical user interface for R (The R Foundation for Statistical Computing, Vienna, Austria, version 3.5.1). This interface is a modified version of R commander (version 2.5–1), which includes statistical functions that are frequently used in biostatistics.

This study adheres to the STROBE reporting recommendations of the Equator Network.

## Results

### Patient background

Patient characteristics are shown in Table 1. The edge of the tumor was below PR in 99.6% of the patients. LLND was performed bilaterally in 90.1% of the patients and unilaterally in 9.9%. There were 89 patients (18.1%) that had pathologically positive lateral lymph nodes. Postoperative adjuvant chemotherapy was administered to 51.4% of the patients.

**Table 1:** Patient characteristics

		*N* = 493 (%)
Age, median (range)		64 (27–85)^a^
Sex	Male	339 (68.8)
	Female	154 (31.2)
Clinical T stage	cTis/ cT1/ cT2/ cT3/ cT4	30/ 422/ 41
Clinical N stage	cN0/ cN1/ cN2	245/ 174/ 74
Distal edge of tumor	Above PR	2 (0.4)
	Below PR	459 (93.1)
	Anal canal	32 (6.5)
Approach	Open	387 (78.5)
	Laparoscopic	55 (11.2)
	Robotic	51 (10.3)
Operative procedure	LAR	255 (51.7)
	APR	226 (45.8)
	ISR	4 (0.8)
	TPE	1 (0.2)
	Hartmann’s procedure	7 (1.4)
LLND	Bilateral LLND	444 (90.1)
	Unilateral LLND, Right/ Left	19 (3.9)/ 30 (6.1)
Pathological T stage	pTis/ pT1/ pT2/ pT3/ pT4	106/ 339/ 48
Pathological N stage	pN0/ pN1/ pN2	236/ 142/ 115
Histological type	Well or Mod^b^	438 (93.6)
	Others	30 (6.4)
Lymphatic invasion	Absent	231 (48.8)
	Present	242 (51.2)
Vascular invasion	Absent	43 (9.9)
	Present	390 (90.1)
LLN metastasis	Positive	89 (18.1)
Adjuvant chemotherapy	Absent	222 (48.6)
	Present	235 (51.4)

*LLN* lateral lymph node, *PR* peritoneal reflection, *LAR* low anterior resection, *APR* abdominoperineal resection, *ISR* intersphincteric resection, *TPE* total pelvic exenteration, *LLND* lateral lymph node dissection

^a^Data are presented as the median (range)

^b^Well or moderately differentiated adenocarcinoma

### Oncological outcome

**Fig. 1:**
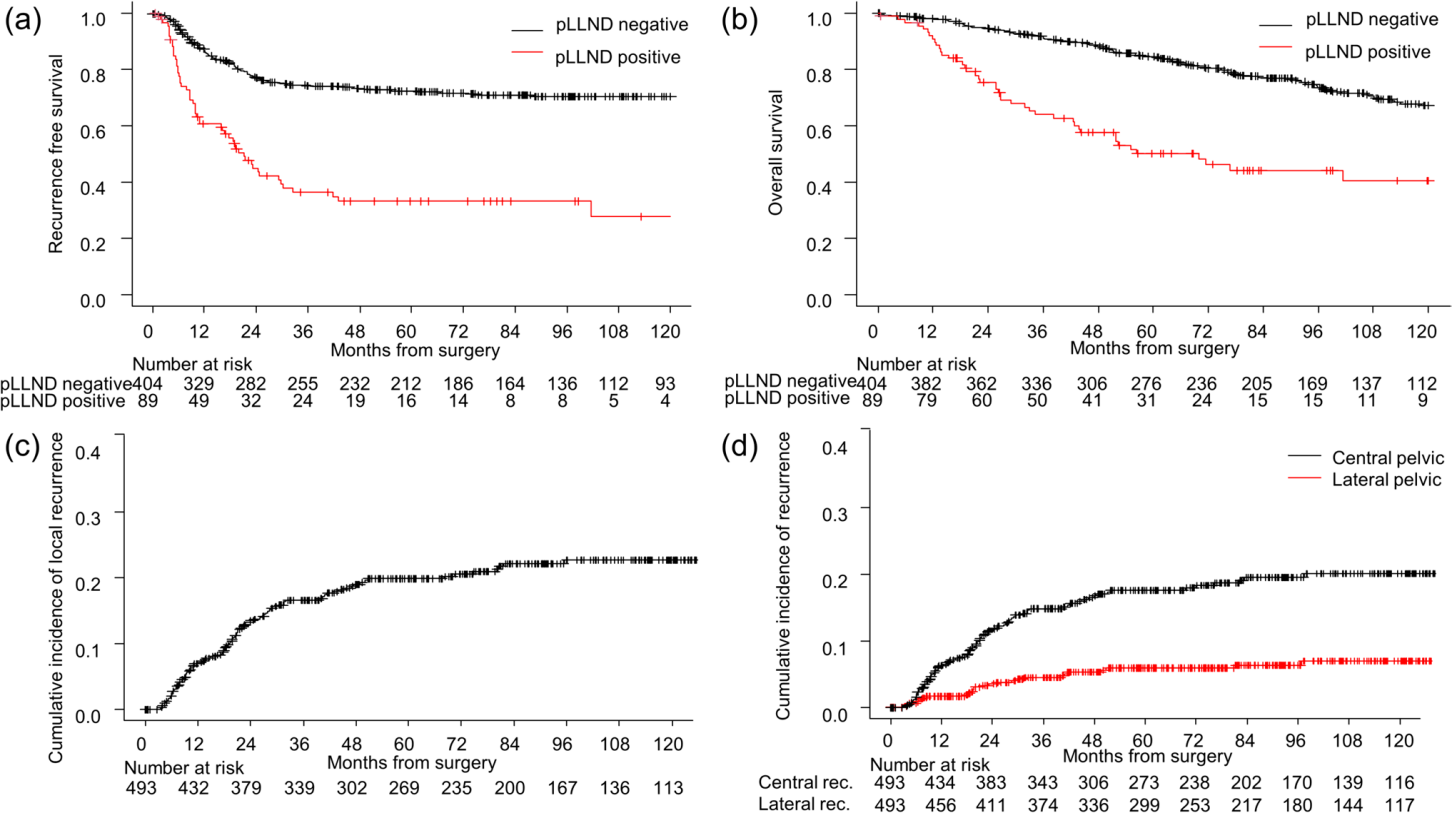
Survival outcome in patients with rectal cancer. Relapse-free (**a**) and overall (**b**) survival rates of patients with (red lines) and without (black lines) pathological lateral lymph node metastasis. Cumulative incidence of local (**c**), central (**d**, black line), and lateral (**d**, red line) recurrences

The median follow-up was 81.2 months (range 4–212.9 months). Figure 1 shows the RFS (a) and OS (b). The 5-year RFS rates were 33.1% and 72.3% in patients with and without pathological LLN metastases, respectively (*p* < 0.01). The 5-year OS rates were 50.0% and 84.9% for patients with and without pathological LLN metastases, respectively (*p* < 0.01).

### LPR after LLND

Local recurrences developed in 97 patients, with a 5-year cumulative incidence of 20.1% (Fig. 1c), and LPR and CPR were observed in 27 and 85 patients after LLND, with 5-year cumulative incidence of 6.7% and 17.4%, respectively (Fig. 1d).

Univariate and multivariate analyses for the risk of LPR are shown in Table 2. In the univariate analysis, seven variables showed a significant correlation with LPR. Multivariate analysis showed that three of these variables were independently associated with LPR: age > 75 years, LLN metastasis, and adjuvant chemotherapy.

**Table 2:** Univariate and multivariate analyses according to the lateral recurrence after lateral pelvic lymph node dissection

		Univariate analysis			Multivariate analysis		
	Variables	5-year LRFS rate	95% CI	*p*-value	HR	95% CI	*p*-value
Age	< 75 years	0.95	0.92–0.98	0.015	1		
	≥ 75 years	0.85	0.69–0.93		5.87	1.94–17.72	< 0.01
Sex	Male	0.94	0.90–0.96	0.57			
	Female	0.95	0.89–0.98				
BMI	< 25	0.93	0.90–0.95	0.24			
	≥ 25	0.98	0.92–0.99				
Approach	Open	0.93	0.90–0.95	0.072	8.22	1.00–67.44	0.023
	Laparoscopic or robotic	0.98	0.89–0.99		1		
LLND	Bilateral	0.93	0.90–0.96	0.24			
	Unilateral	1.00	NA–NA				
pT stage	Tis/T1/T2	0.92	0.85–0.96	0.58			
	T3/T4	0.95	0.91–0.97				
Tumor size	< 50 mm	0.96	0.92–0.98	0.13			
	≥ 50 mm	0.92	0.88–0.95				
pLLN metastasis	Absent	0.96	0.94–0.98	< 0.01	1		
	Present	0.79	0.65–0.87		3.80	1.45–9.99	< 0.01
pMLN metastasis	Absent	0.96	0.93–0.98	0.021			
	Present	0.90	0.85–0.94		0.65	0.23–1.89	0.43
Histological	Well/Mod^a^	0.94	0.92–0.97	< 0.01	1		
	Others	0.85	0.64–0.94		0.39	0.14–1.06	0.065
Lymphatic invasion	Absent	0.96	0.92–0.98	0.20			
	Present	0.92	0.87–0.95				
Vascular invasion	Absent	0.97	0.84–0.99	0.32			
	Present	0.94	0.90–0.96				
Adj. CT	Absent	0.98	0.95–0.99	< 0.01	1		
	Present	0.89	0.84–0.93		7.51	1.85–30.45	< 0.01
Anal preservation	Yes	0.97	0.94–0.98	< 0.01	1		
	No	0.90	0.85–0.93		2.96	1.16–7.57	0.023

*BMI* body mass index, *pT* pathological T, *pLLN* pathological lateral lymph node, *pMLN* pathological mesorectal lymph node, *Adj* adjuvant, *CT* chemotherapy, *LRFS* lateral recurrence-free survival, *HR* hazard ratio, *CI* confidence interval, *NA* not available

^a^Well or moderately differentiated adenocarcinoma

**Fig. 2:**
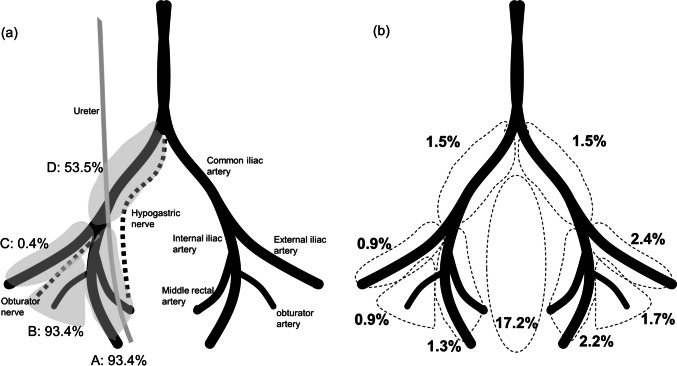
Percentage of the dissected lateral pelvic areas and incidence of recurrence in each area. **a** Proportion of the patients who underwent complete removal of the lymph nodes in each area. A: internal iliac, B: obturator, C: external iliac, and D: common iliac nodes. **b** The distribution and frequency of local recurrences are shown. In the lateral areas, the percentage of patients who developed recurrence in each area (area ABCD) among those who underwent a complete dissection of the area is presented. In the central pelvis, the percentage of patients who developed central pelvic recurrence is presented because all patients underwent total mesorectal excision

The locations and percentages of local recurrences are shown in Fig. 2. The internal iliac (A) and obturator (B) areas were routinely dissected because of the high frequency of metastases. Systematic complete lymph node dissection was performed in 93.4% of area A and 93.4% of area B, although not in all patients because of unilateral dissection in some cases (Fig. 2a). Recurrence rates after LLND in each area are presented in Fig. 2b. Because there were cases of unilateral dissection, the frequencies were calculated based on the number of cases with dissection on each side. On the right side, the incidence of recurrence was 1.3%, 0.9%, 0.9%, and 1.5% in areas A, B, C, and D, respectively. On the left side, the incidence of recurrence was 2.2%, 1.7%, 2.4%, and 1.5% in areas A, B, C, and D, respectively. A CPR of 17.2% was observed in all patients.

### Case presentation

**Fig. 3:**
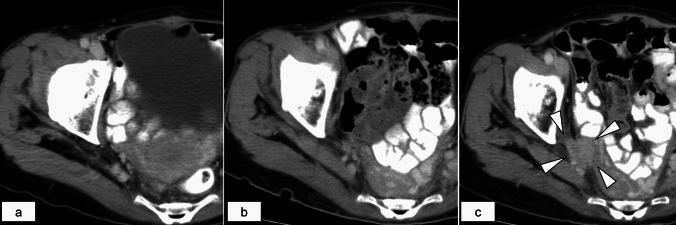
A representative case of lateral pelvic recurrence after lateral pelvic lymph node dissection. Computed tomography images obtained preoperatively (**a**), 3 months after surgery (**b**), and 2 years after surgery (**c**) are shown. The white triangles indicate recurrent nodules

A representative case of local recurrence after LLND is shown (Fig. 3). A 68-year-old woman with lower rectal cancer underwent abdominoperineal resection and bilateral LLND without preoperative treatment. The final pathological diagnosis revealed stage I (T2N0M0) with the circumferential margin negative, and no metastasis was present in the dissected lateral lymph nodes on both the right and left sides. The preoperative CT image is shown in Fig. 3a, and the first postoperative CT image is shown in Fig. 3b. During surveillance without adjuvant chemotherapy, lymph node recurrence in the right obturator area was observed using CT at 24 months after surgery (Fig. 3c).

## Discussion and conclusions

This study is the first comprehensive examination of recurrence after LLND without prior treatment, utilizing a substantial cohort of patients with advanced lower rectal cancer. In this study, TME plus bilateral LLND was performed in 90% of cases because this was the standard procedure in Japan during the study period and is recommended in the Japanese treatment guidelines for colorectal cancer published in 2005 [15]. Because preoperative treatments for rectal cancer, such as CRT or total neoadjuvant therapy, are not recommended in the guidelines, preoperative treatment was performed none of the patients in the present study.

We initially evaluated the prognosis of patients with LLND. Among patients who underwent LLND for Stage II/III rectal cancer, 20% had positive LLN metastases, and the 5-year RFS and OS rates were 33.1% and 50.0%, respectively. In previous studies, the 3-year OS and RFS rates in patients with rectal cancer and LLN metastasis were reportedly 37–60% and 37–39%, respectively, similar to our results. Although the prognosis of patients with LLN metastasis is poor, approximately half of the patients survived for 5 years without an impaired quality of life due to increased metastasis in the pelvic sidewall by the additional LLND.

In the large-scale randomized trial JCOG0212, which investigated the impact of LLND in a cohort without obvious LLN metastasis without pretreatment, recurrence in the pelvic sidewall decreased from 7 to 2% with the addition of LLND [13]. The lateral recurrence rate after LLND was 7% in the present study, which was higher than that reported by JCOG0212, probably because our cohort included many cases with suspected LLN metastasis in preoperative imaging modalities. In our multivariate analysis, the presence of LLN metastasis was a risk factor for lateral recurrence, probably due to residual micrometastasis in the dissected or undissected lateral area, such as the common iliac nodes. The results of JCOG0212 demonstrated that LLND decreased lateral recurrence but not central recurrence [16], whereas CRT without LLND has been reported to decrease local recurrence in total; however, lateral recurrence also reportedly remained a major local recurrence site even after CRT [17, 18]. To compensate for the drawbacks of these treatments, a combination of CRT and with LLND in selected patients has recently been considered a novel treatment option. Several studies have demonstrated favorable outcomes with this combination strategy [17, 19, 20].

This is the first study to investigate the site of lateral recurrence after LLND. As shown in Fig. 2, the recurrence rate in the dissected area was approximately 1%, regardless of the location. The overall lateral recurrence rate was 5%, and this discrepancy was partly due to the recurrence in undissected lateral areas. In this study, dissection of the internal iliac and obturator nodes was routinely performed. However, other lateral nodes, including the external and common iliac nodes, were dissected only in cases with suspected metastases in these areas. Considering the relatively low incidence of metastasis in these areas [21, 22], the complexity of dissecting these areas [23], and the fact that LLN metastasis in those area has a poor prognosis equivalent to systemic disease [24, 25], prophylactic extended resection of nodes in these areas should be determined cautiously.

As in the present case, the deepest area connecting the internal iliac and obturator areas contains complex nerve and vascular structures, making it difficult to perform a complete dissection of the small nodes, and consequently developing recurrences even after lymph node dissection. Several recent reports have demonstrated a larger number of dissected lymph nodes or better prognostic outcomes after robotic LLND than after laparoscopic surgery [26–28]. The use of indocyanine green (ICG) fluorescence in robotic LLND has been reported to result in a 3-year cumulative lateral recurrence rate of 0% [29], suggesting that the outcomes after LLND in our series could have been improved by these techniques because our series included a relatively small number of robotic cases and no cases of ICG application.

The present study had several limitations. First, the study period was relatively long and, therefore, included a wide range of treatments, such as open, laparoscopic, or robotic surgery, and different indications for adjuvant chemotherapy. Because the majority of LLND are performed robotically, further updates of prognostic outcomes are necessary in the future. Second, the quality of LLND largely differs according to the technique used at each institution. As this study was conducted in a Japanese high-volume cancer center in which LLND was routinely performed, the outcomes in other institutions or countries that are not so accustomed to LLND could not be as good.

In conclusion, we demonstrated the prognostic outcomes and limitations of LLND for patients with advanced lower rectal cancer, focusing on the incidence of recurrence in the lateral area after LLND. LLND is an essential technique that surgeons should acquire.

## Data Availability

No datasets were generated or analysed during the current study.
